# A Retrospective, Unicentric Evaluation of Complicated Diverticulosis Jejuni: Symptoms, Treatment, and Postoperative Course

**DOI:** 10.3389/fsurg.2015.00057

**Published:** 2015-11-13

**Authors:** Patrick Téoule, Emrullah Birgin, Benjamin Zaltenbach, Georg Kähler, Torsten J. Wilhelm, Peter Kienle, Felix Rückert

**Affiliations:** ^1^Department of Surgery, Medical Faculty Mannheim, University Medical Centre Mannheim, Heidelberg University, Mannheim, Germany

**Keywords:** complicated jejunal diverticulitis, perioperative management, acute abdomen, visceral surgery, rare disease

## Abstract

**Background:**

In contrast to the diverticulosis of the colon, jejunal diverticulosis is a rare condition. The incidence is 0.06–5% in large autopsy series. Complicated diverticulosis jejuni (CDJ) often presents with unspecific symptoms. Therefore, diagnosis is often a challenging process and due to the clinical rarity generally valid recommendation of perioperative management does not exist.

**Patients and methods:**

We considered only patients who were operated in our center between April 2007 and August 2014. Patients were identified by data bank search via International Statistical Classification of Diseases and Related Health Problems diagnosis code K57.10. Data were manually screened, and patients with Meckel’s and duodenal diverticula were excluded from this study. Eleven consecutive patients with CDJ were finally included in this study. We analyzed symptoms, diagnostic procedures, surgical treatment, and postoperative morbidity and mortality.

**Results:**

The median age of our patients was 76 years (range: 34–87). CDJ presented most frequently as intestinal bleeding or as diverticulitis. Clinical symptoms were unspecific abdominal pain, hematemesis or melena, ileus, nausea, and emesis as well as patients with acute abdomen. Esophagogastroduodenoscopies confirmed CDJ in two of the three patients. An abdominal computed tomography scan only helped to diagnose CDJ in two of the 10 patients. Eight (72.7%) patients received an open segmental resection with primary anastomosis. In three (27.3%) cases, a reoperation was necessary. Overall morbidity rate was 45.5%, and perioperative mortality was 9.1%.

**Conclusion:**

Due to the acute character of the disease, patients with CDJ are seriously ill. To diagnose patients with CDJ remains challenging as diagnostic investigations are usually not helpful in confirming the diagnosis. Still, diagnosis of CDJ is most frequently confirmed intraoperatively.

## Introduction

In contrast to the diverticulosis of the colon, jejunal diverticulosis is a rare disease. Incidence ranges from 0.06 to –5% in large autopsy series (1, 2). Most of the patients with jejunal diverticulosis are asymptomatic throughout their live. Some of the patients with diverticulitis might present with unspecific problems as intermittent abdominal pain, emesis, constipation, and diarrhea (3). Other patients will experience complications like bleeding or perforation, as in diverticulosis of the colon. The complicated diverticulosis jejuni (CDJ) is a very rare disease. Only 10–30% of the patients show such complications (4). In contrast to the diverticulosis of the colon that can often be diagnosed by its typical clinical presentation and symptoms, the CDJ displays unspecific symptoms. This is due to the relatively variable anatomic location of the small bowel. Previous studies could not identify typical symptoms in these patients that might result in further specific diagnostic and therapy (5, 6). However, such early diagnostic might be favorable as perforation or bleeding can result in severe patients’ condition. Such a complication can result in a mortality rate in previously published articles that ranges between 24 and 40% (7–9). The aim of this retrospective study was to retrospectively evaluate a cohort of patients with CDJ in a single center for colorectal surgery. The typical symptoms and findings of medical imaging were analyzed. By this, we hope to improve the perioperative management in the sense of evidence-based medicine. A secondary aim was to analyze and discuss the operative strategies and short-term results in patients with CDJ.

## Patients and Methods

### Patients

Medical and operative reports of all patients treated for CDJ at the department of Surgery, University Medical Centre Mannheim, Medical Faculty Mannheim, Heidelberg University, during the period from April 2007 to August 2014 were analyzed retrospectively. CDJ was defined as inflamed diverticula *ante perforationem*, free or sealed perforation, and bleeding diverticula requiring surgery. Meckel’s and duodenal diverticula were excluded. All included patients underwent surgery. Ethical approval for the retrospective analysis was obtained from the local ethics committee. The study was registered at www.researchregistry.com (UIN researchregistry573) and approved by the local ethics committee. All patients finally received end-to-end jejunojejunostomy after resection of the affected jejunal part. A single-layer anastomosis was created with running suture using PDS 4/0.

### Data Collection and Statistics

Demographic characteristics, such as gender and age, American Society of Anaesthesiologists (ASA) status, and comorbidities as well as body mass index (BMI) were analyzed. We focused on the preoperative symptoms and the non-clinical diagnostics with respective to their predictive value, operative strategies, postoperative events and outcomes, as well as in-hospital mortality. The predictive value in percentage for a given diagnostic tool was defined as number of congruent preoperative and intraoperative finding divided by the number of all performed examinations. Postoperative complications were graded according to the Clavien–Dindo classification (10). Patient’s characteristics and parameters used for statistical analysis are listed in the Supplementary Material. After resection, patients were routinely observed at an intermediate care unit (IMC). Statistical analysis was performed by SPSS 15.0 (SPSS, Inc., Chicago, IL, USA) statistical software. All clinical and pathological characteristics were stratified to build categorical or nominal variables.

## Results

### Demographics and Clinical Data

Eleven patients with CDJ, four men (36.4%) and seven women (63.6%), were treated and underwent surgery at our department over a 7-year period. Median age of these patients was 76 (range: 34–87; Table 1).

**Table 1:** **Patient cohort demographic and clinical data**.

	*n* = 11 (%)
Male sex	4 (36.4)
Age, median (range) (years)	76 (34–87)
Hypertension	5 (45.5)
Atrial fibrillation	4 (36.4)
Coronary heart disease	3 (27.3)
Others^a^	6 (54.5)
BMI (range) (kg/m^2^)	25.4 (22–27)
ASA status
1	0
2	4 (36.4)
3	2 (18.2)
4	1 (9.1)
x	4 (36.4)

*Results of patient’s characteristics in 11 patients with complicated diverticulosis jejuni*.

*ASA, American Society of Anaesthesiologists; BMI, body mass index; x is missing data*.

*^a^Multiple answers are possible; others are diabetes mellitus, chronic obstructive pulmonary disease, chronic renal failure, acute myeloid leukemia, and epilepsy*.

### Symptoms and Diagnostic Procedures

The most frequent and leading clinical manifestation of our patient cohort of eleven patients was abdominal pain in eight cases (72.7%). Four of these patients (50.0%) presented in the sense of acute abdomen (patient Nos. 2, 6, 7, and 10). Less frequent symptoms were gastrointestinal hemorrhage in three cases (27.3%; Nos. 4, 7, and 8), ileus in two cases (18.2%; Nos. 5 and 11), and nausea and emesis in two cases (18.2%; Nos. 3 and 9). Abdominal ultrasound was performed in nine (81.8%) patients, esophagogastroduodenoscopy (EGD) was performed in three (27.3%) patients, and colonoscopy was performed in two (18.2%) patients. An abdominal computed tomography (CT) was performed in 10 (90.9%) patients. Table 2 summarizes the duration of complaints (acute or subclinical), the kind of performed examination, and their results. In Table 3, the predictive value of the non-clinical diagnostics is indicated. Two (66.7%) out of three performed EGD helped in medical decision making. In one patient, a diverticula bleeding could initially be clipped successfully (no. 8). The endoscopy of the other patient showed blood distal from the ligament of Treitz (No. 4). Eight of the 10 (80%) patients performed abdominal CT scans and showed unspecific findings that were not helpful in the diagnosis of CDJ. In two patients, the CT findings were conclusive. In the first patients, CT finding listed a sealed perforation from the small bowel (No. 6) and in the second a bleeding after endoscopic clipping (No. 8; Tables 2 and 3).

**Table 2:** **Preoperative findings and diagnostics**.

Patient	Symptoms (duration)	Apparative diagnostics	Preop. diagnosis	Intraoperative findings	Procedure	Reoperation	Findings reoperation
1	Abdominal pain LIF + P-U (2 days)	Ultrasound and abdominal CT scan	Small bowel conglomeration LIF	CDJ 20–80 cm after Treitz with sealed perforation and LP	DL and SR (60 cm)	None	None
2	Diffuse abdominal pain (acute)	Ultrasound and abdominal CT scan	Hollow organ perforation	CDJ 40 cm after Treitz with multiple perforation, abscess, and LP	DCS and SR (80 cm)	SL (1 day later) + anastomosis	Two blind stapled ends, without peritonitis
3	Abdominal pain LIF + N and V (2 days)	Ultrasound and abdominal CT scan	Small bowel wall thickening	UDJ 70 cm after Treitz without perforation or LP	DL, EL and lavage	SL (2 days later), perforated diverticula SR (10 cm)	Suspected microperforation 70 cm after Treitz
4	Gastrointestinal hemorrhage (several days)	EGD, CC, ultrasound, and abdominal CT	Gastrointestinal bleeding and small bowel conglomeration LIF	CDJ 50 cm after Treitz with blood-filled diverticula	ES and SR (20 cm)	None	None
5	Ileus (1 day)	Ultrasound and abdominal CT scan	Suspected mesenterial ischemia	CDJ 60 cm after Treitz with perforation and torsion	SR (10 cm)	None	None
6	Abdominal pain LUQ and LIF (acute)	Ultrasound and abdominal CT scan	Sealed perforation LIF and possibly small bowel	Small bowel conglomeration with CDJ and interenteric abscess	Adhesiolysis and SR (10 cm)	None	None
7	Diffuse abdominal pain (acute) and gastrointestinal hemorrhage	Ultrasound, EGD, CC, and abdominal CT scan	No conclusive findings	4Q-peritonitis with UDJ	DL, EL, and lavage	Abscess drainage (18 days later), SR (20 cm)	Burst abdomen, CDJ 30 cm after Treitz with sealed perforation and tubo-ovarian abscess
8	Gastrointestinal hemorrhage (several days)	EGD, CC, and abdominal CT angiography	Upper gastrointestinal hemorrhage	CDJ 50 cm after Treitz, with clipped and blood-filled diverticula	SR (20 cm)	None	None
9	Diffuse abdominal pain, N and V (3 days)	Ultrasound	Small bowel wall thickening	CDJ directly and 90 cm after Treitz, 4Q-peritonitis and perforation	DL and SR (10 + 5 cm)	None	None
10	Abdominal pain LIF (acute)	Ultrasound and abdominal CT scan	Suspicion of mesenteric infarction	CDJ 50 cm after Treitz ante perforationem	DL and SR (25 cm)	None	None
11	Diffuse abdominal pain and ileus (2 days)	Ultrasound and abdominal CT scan	Small bowel wall thickening and ileus	CDJ 20 cm after Treitz ante perforationem, abscess, and LP	DL and SR (10 cm)	None	None

*Results of preoperative findings and diagnostics as well as operative treatment in 11 patients with complicated diverticulosis jejuni*.

*LIF, left iliac fossa; LUQ, left upper quadrant; P-U, periumbilical; CDJ, complicated diverticulosis jejuni; UDJ, uncomplicated diverticulosis jejuni; LP, local peritonitis; N and V, nausea and vomiting; DL, diagnostic laparoscopy; EL, exploratory laparotomy; SR, open segment resection and anastomosis; DCS, damage control surgery; SL, second look operation; ES, on table enteroscopy; EGD, esophagogastroduodenoscopy; CC, coloscopy*.

**Table 3:** **Preoperative symptoms and predictive value of apparative diagnostics**.

	*n* = 11 (%)
**Preoperative symptoms^a^**
Abdominal pain	8 (72.7)
Upper gastrointestinal bleeding/melena	3 (27.3)
Ileus	2 (18.2)
Nausea/emesis	2 (18.2)
**Preoperative diagnostic**
Endoscopic diagnostic	3 (27.3)
Positive finding	2 (66.7)
Abdominal computed tomography	10 (90.9)
Positive finding	2 (20.0)

*Results of clinical symptoms and predictive value of apparative diagnostics in 11 patients with complicated diverticulosis jejuni*.

*^a^Multiple answers are possible*.

### Intraoperative Findings and Operative Treatment

Perforated CDJ (Nos. 2, 5, and 9) and sealed perforation (Nos. 1, 6, and 7) were observed each in three (27.3%) patients. One (9.1%) microperforation was noted (No. 3). Bleeding diverticula (Nos. 4 and 8) and inflamed *diverticula ante perforationem* (Nos. 10 and 11) were found each in two (18.2%) cases.

**Figure 1:**
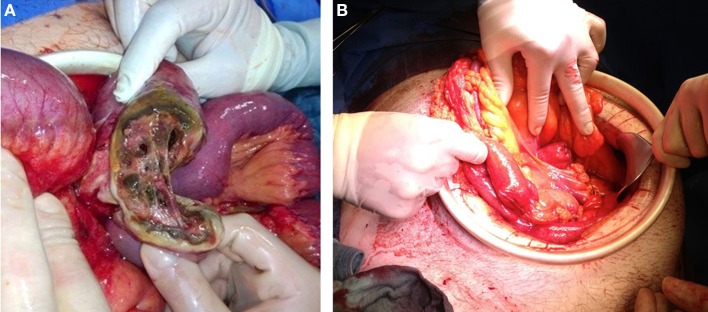
**Two pictures after laparotomy of one patient with perforated diverticulosis jejuni and acute myeloid leukemia**. [**(A)** before resection and **(B)** after damage control surgery with two blind stapled ends].

**Figure 2:**
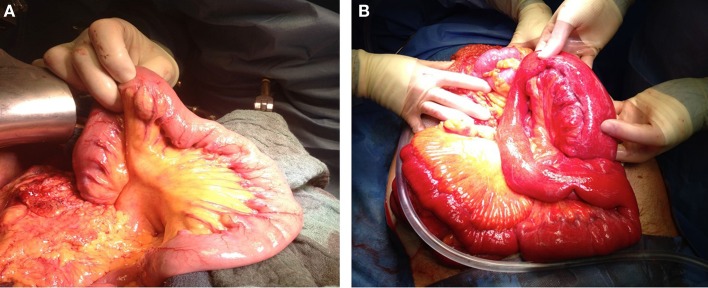
**Two pictures after laparotomy of two patients with jejunal diverticulosis [(A) uncomplicated diverticulosis jejuni and (B) complicated diverticulosis jejuni]**.

Median of operative time was 125 min (range: 78–255), and median blood loss was 100 ml (range: 50–500). The surgical approach was individual and dependent on the intraoperative finding. Eight (72.7%) patients received an open segment resection with primary anastomosis (Nos. 4–6 and 8). One of the patients (9.1%) received an initial damage control surgery with discontinuity resection and lavage. This was due to the patients’ instable condition after chemotherapy for acute lymphatic leukemia. The first operation was followed by a second look operation on the next day with reanastomosis (No. 2; Figure 1). Patient number 3 had an explorative laparotomy. Intraoperatively an uncomplicated diverticulosis jejuni with no sign of perforation was found. After 2 days, a second look operation was performed due to clinical aggravation. During this second operation, a perforated diverticula was diagnosed (Figure 2). In patient number 7, a purulent peritonitis of unknown cause was seen during diagnostic laparoscopy; UJD was noticed. After conversion laparotomy, the operation was terminated with extensive lavage. Initially, the patient recovered well. However, after 18 days a reoperation had to be performed due to a burst abdomen and clinical aggravation. A CDJ 30 cm after the ligament of Treitz was found with a sealed perforation along with a tubo-ovarian abscess as secondary finding. In both of the abovementioned cases (patient Nos. 3 and 7), a resection of the affected jejunal part, reanastomosis, extensive lavage, and drainage was performed. Table 2 summarizes the intraoperative finding, especially the location of the resected jejunal section and the length of resected small bowel, as well as the operative strategies.

### Morbidity and Mortality

Postoperative complications occurred in five (45.5%) patients. Three (27.3%) of our patients had only one complication, one patient (9.1%) developed two complications, and one patient (9.1%) had four complications. In declining order, wound infections (three cases, 27.3%; Nos. 2, 5, and 9), urinary retention (one case, 9.1%; No. 3), and multiorgan failure due to sepsis (one case, 9.1%; No. 7) could be observed (Table 4).

**Table 4:** **Morbidity and mortality**.

	*n* = 11 (%)
Patients with complication^a^	5 (45.5)
Clavien-Grade I	4 (36.4)
Clavien-Grade V	1 (9.1)
Reoperation	3 (27.3)
LOS, median (range) (days)
Intermediate care	2 (1–19)
Overall duration	10 (7–27)
In-hospital mortality	1 (9.1)

*Results of postoperative characteristics in 11 patients with complicated diverticulosis jejuni*.

*^a^Multiple answers are possible; MOF, multiorgan failure; LOS, length of stay; IMC, intermediate care*.

A reoperation was required in three cases (27.3%): two of the three patients received a planned relaparotomy (Nos. 2 and 3) and one patient was reoperated due to a burst abdomen and clinical aggravation, as mentioned earlier (No. 7). One (9.1%) of our 11 patients died within the postoperative course due to multiorgan failure caused by sepsis (No. 7). This patient was a 76-year-old multimorbid woman. The patient had a preexisting chronic obstructive pulmonary disease, chronic renal failure, and history of myocardial infarction. The median of postoperative length of stay in our patient cohort was 10 days (range: 7–27), whereas the median of length of stay on Intermediate Care (IMC) was 2 days (range: 1–19).

## Discussion

Complicated diverticulosis jejuni is a very rare disease. The first reports of jejunal diverticulosis were in 1794 by Somerling et al. and in 1807 by Cooper et al. (11). In 1906, Gordinier and Sampson described the first patient who underwent an operation due to jejunal diverticulosis (12). Jejunal diverticulosis is usually a silent disorder, with unspecific symptoms, until it presents with acute complications. Complications requiring a surgical intervention occur in up to 30% of patients (13). These include gastrointestinal obstruction and hemorrhage as well as perforation (6, 8, 14–17). Pain is an important symptom for perforation or abscess in patients with CDJ (18). In our patient cohort, this symptom was observed in seven (63.6%) patients with peritonitis or intra-abdominal abscess. Additional one patient with severe inflammation had abdominal pain. Therefore, pain indeed seems to be an important finding in CDJ. However, the character of the pain was mostly unspecific. Additional, as a result of the varying location of jejunal diverticula, there is no characteristic localization of pain in patients with CDJ. In comparison to other intra-abdominal acute conditions such as appendicitis, cholecystitis, or colonic diverticulitis, there are no typical clinical findings that might confirm the presence of CDJ. These facts relativize the clinical importance. Clinicians should be aware that a less severe form of CDJ with only mild pain might remain undetected. Therefore, CDJ should be kept in mind as rare differential diagnosis in patient with abdominal pain. Three of our patients (27.3%) presented with hemorrhage, another manifestation of CDJ (17). It is difficult to compare this number with previous reports, as these differ widely in the observed patient cohorts. As endoscopy of the small bowel is not easy to perform, this symptom is difficult to assess.

In our opinion, pains as well as hemorrhage seem to be too unspecific to help in the early diagnosis of CDJ. However, CDJ should be kept in mind as differential diagnosis in patient presenting with these symptoms.

Non-clinical diagnostics such as abdominal CT, which usually detects or confirms a suspected preoperative diagnosis in other clinical pictures, can only reveal unspecific findings in CDJ. These findings include entrapped air or imbibition of fat tissue within the small bowel (19). In our series, only two out of 10 performed abdominal computed tomographies (20%) were helpful in the diagnosis of CDJ. Although previous studies showed that a CT scan might be helpful, our data do not support this. However, it is an important tool to rule out other differential diagnoses. In our cohort, explorative laparoscopy was the definitive diagnostic procedure in confirming CDJ. The treatment of most of our patients was determined based on the findings during laparoscopy. In synopsis, CDJ is a very rare disease with varying locations and symptoms. Today, clinical examination and anamnesis still remain the critical factor in setting the indication for surgical exploration. However, due to its rareness, CDJ is still a diagnosis by exclusion. If the diagnosis is fixed, the choice of operative method should be based on the principles of septic surgery implying immediate decontamination and healthy wound edges. Surgical resection of the involved jejunal section and primary anastomosis is the treatment of choice (7, 17, 20–22). In our patient cohort, eight (72.7%) patients had an open segment resection with primary anastomosis. One (9.1%) patient in critical condition underwent an initial damage control surgery with an open segment resection and extensive lavage, followed by a second look operation on the next day with reanastomosis.

As with most other acute diseases, the postoperative course is strongly influenced by previous medical conditions, as well as the extent of intraoperative findings (23). The incidence of CDJ increases with age, with the peak occurring in the sixth and seventh decades of life (6, 8, 20). Elderly patients have more frequently preexisting health problems and are in higher danger of complications (24). In our patient cohort, the median age was 76 years. One patient who died postoperatively was indeed of advanced age with a poor preoperatively health condition.

Out study shows that CDJ still presents formidable challenges in diagnosis and treatment. When therapy is delayed, further complications can lead to life-threatening consequences. Therefore, the emergency surgeon should always consider this differential diagnosis in acute abdomen.

## Conclusion

As a result of its rarity and diffuse symptoms, diagnosis of CDJ remains a challenging process. Despite extensive preoperative diagnostics, definitive diagnosis of CDJ can often only be made intraoperatively. Due to the acute character of the disease, patients are often seriously ill and this should be considered in the perioperative management, especially as this disease is frequent in elderly patients.

## Ethics Statement

This study was approved by the Ethikkomission Mannheim II; the decision included the permission to retrospectively evaluate patients data. Due to the setting of the study, it was stated that written consent was not necessary for the evaluation.

## Authors Contribution

PT, PK, and FR participated in the conception and design of the study. PT and FR performed the research, analyzed the data, and drafted the manuscript. EB and BZ performed data collection. GK and TW performed data evaluation. BZ, GK, and TW participated in the revision of the manuscript. All authors have read and approved the final manuscript.

## Conflict of Interest Statement

The authors report no proprietary or commercial interest in any product mentioned or concept discussed in this article.
