# Supplementary material for: A Retrospective, Unicentric Evaluation of Complicated Diverticulosis Jejuni: Symptoms, Treatment, and Postoperative Course
Source: Front Surg. 2015 Nov 13;2:57. doi: 10.3389/fsurg.2015.00057 (PMC4643121; doi:10.3389/fsurg.2015.00057)
Supplement: Supplementary file 1 [file table_1.doc]

## Supplemental information

SI: Patients characteristic

|  | **parameters** |
| --- | --- |
| Patients characteristics | Sex; age; BMI; hypertension; atrial fibrillation; coronary heart disease; diabetes mellitus; chronic pulmonary disease; chronic renal failure, acute myeloid leukaemia; epilepsy; ASA-status |
| Preoperative symptoms | Abdominal pain, with localization and duration; gastrointestinal haemorrhage; nausea and vomiting; ileus |
| Preoperative diagnostics | Abdominal unltrasound; abdominal computed tomography with or without angiography; esophagogastroduo-denoscopy; coloscopy; |
| Intraoperative findings | Operative time; blood loss; concerned jejunal section; kind of perforation; peritonitis or abscess; |
| Postoperative data | Length ot stay (overall duration and intermediate care); occurred complications; reoperation; in-hospital-mortality |

**SI: Table 1** Patient characteristics and parameters used for statistical analysis

(ASA= American society of anesthesists, BMI= Body-mass-index).
